# Identification of features contributing to outliers in BCF prediction

**DOI:** 10.1093/bib/bbaf631.046

**Published:** 2025-12-12

**Authors:** Riku Maeda, Kei Nakayama, Rumi Tagami, Midori Iida

**Affiliations:** Department of Physics and Information Technology, Kyushu Institute of Technology, Iizuka, Fukuoka, Japan; Center for Marine Environmental Studies, Ehime University, Matsuyama, Japan; Center for Marine Environmental Studies, Ehime University, Matsuyama, Japan; Department of Physics and Information Technology, Kyushu Institute of Technology, Iizuka, Fukuoka, Japan

## Abstract

**Aim:**

The bioconcentration factor (BCF) indicates the potential of chemicals to accumulate in organisms. Greater BCFs imply higher bioaccumulation and health risks [1]. To date, BCF prediction models based on molecular structure have mainly been evaluated for accuracy [2,3], with limited systematic assessment of their reliability or identification of outliers. In this study, we aimed to improve model practicality.

**Methods:**

We developed a regression model based on physicochemical descriptors from SMILES strings and compared predicted BCF values with experimental data. We then constructed a classifier to identify chemicals with large prediction deviations (overestimation and underestimation groups) and analyzed the contribution of individual features.

**Results:**

In the underestimation group, electrical properties (qnmax), hydrophobicity (LogP), and the presence or absence of specific functional groups strongly influenced predictions. In contrast, no interpretable patterns in important molecular descriptors were identified in the overestimated group.

**Conclusion:**

These findings provide insights into the chemicals for which BCF predictions should be interpreted with caution and may contribute to more efficient environmental risk assessments.

**References:**

1. Arnot J.A., Gobas F.A.P.C. ‘A review of bioconcentration factor (BCF) and bioaccumulation factor (BAF) assessments for organic chemicals in aquatic organisms.’ *Environmental Reviews* 2006:14(4).

2. Kobayashi Y., Yoshida K. ‘Development of QSAR models for prediction of fish bioconcentration factors using physicochemical properties and molecular descriptors with machine learning algorithms.’ *Ecological Informatics* 2021: 63:101285.

3. Pore S. et al. ‘Machine learning-based q-RASAR predictions of the bioconcentration factor of organic molecules estimated following the organisation for economic co-operation and development guideline 305.’ *Journal of Hazardous Materials* 2024: 479:135725.

